# Antimicrobial activity of flavonoids from *Sedum aizoon* L. against *Aeromonas* in culture medium and in frozen pork

**DOI:** 10.1002/fsn3.1178

**Published:** 2019-09-01

**Authors:** Feng Xu, Shifeng Cao, Chunxing Wang, Kaikai Wang, Yingying Wei, Xingfeng Shao, Hongfei Wang

**Affiliations:** ^1^ State Key Laboratory for Quality and Safety of Agro‐products, Key Laboratory of Animal Protein Food Processing Technology of Zhejiang Province, College of Food and Pharmaceutical Sciences Ningbo University Ningbo China; ^2^ College of Biological and Environmental Sciences, Key Laboratory of Fruits and Vegetables Postharvest and Processing Technology Research of Zhejiang Province Zhejiang Wanli University Ningbo China

**Keywords:** *Aeromonas*, antimicrobial activity, flavonoids, pork meat, *Sedum aizoon* L.

## Abstract

The chemical composition and antimicrobial activity of flavonoids from *Sedum aizoon* L. against *Aeromonas *in vitro were investigated, and the effect of flavonoid treatment on the quality of fresh pork during frozen storage for 6 months was also explored. The results showed that kaempferol, quercetin dihydrate, and catechin were the most predominant flavonoids from *S. aizoon* L. Flavonoids exhibited antibacterial activity to *Aeromonas *in vitro, which caused membrane damage, disruption of the bacterial surface, and internal ultrastructure, and resulted in the leakage of reducing sugars and proteins. Meanwhile, flavonoid treatment retarded the microbial growth and deteriorates of pork characteristics, including pH value, total volatile basic nitrogen (TVB‐N), texture, and sensory evaluation during frozen storage, thereby prolonged the shelf life. Their results suggested that flavonoids from *S. aizoon* L. offer a promising choice for food safety and preservation.

## 1 INTRODUCTION

The corruption of frozen pork caused by microorganism is still the severest economic issues facing the food industry (Diao, Hu, Zhang, & Xu, 2014; Sokmen et al., 2004). It has been reported that the initial composition of bacterial flora and the method of preservation are the main factors determining the shelf life of pork. *Aeromonas* are thought as the main group of microbes isolated from pork (Palumbo, 1988). To extend the shelf life, some synthetic additives were used to inhibit microorganisms. However, considering the safety of synthetic chemicals, much attention has been paid to use natural antimicrobial materials for food preservation to extend the shelf life of meat (Kamboh, Memon, Mughal, Memon, & Bakhetgul, 2018) such as natural extracts from tea, grape, chestnut, seaweed (Lorenzo, Sineiro, Amado, & Franco, 2014), and natural antimicrobial substance from lactic acid bacteria (Miao et al., 2015).

*Sedum aizoon* L. (*Crassulaceae Sedum*) is an important edible and medicinal plant in China and has a positively effective on preventing some diseases like elevated hematic fat and hypertension (Xu et al., 2015). A few biological components have been isolated from this plant genus, including flavonoids, phenolic acid compounds, polysaccharose, and alkaloids (Stevens, Hart, Elema, & Bolck, 1996). Our previous work (2018) found that flavonoids from *S. aizoon* L. could enhance the shelf life of refrigerated pork and show antibacterial activity to lactic acid bacteria in vitro. Nonetheless, the information about the application of flavonoids from *S. aizoon* L. and their antimicrobial activity against *Aeromonas* is limited.

This paper aims to identify the flavonoid components in *S. aizoon* L. using high‐performance liquid chromatography (HPLC), and the antibacterial activity against *Aeromonas *in vitro of the flavonoids from *S. aizoon* L. was investigated. Furthermore, the effect of flavonoids on the quality of frozen pork was also analyzed.

## 2 MATERIALS AND METHODS

### 2.1 Plant material

*S. aizoon* L. were harvested from a plantation in Ningbo University (Zhejiang Province, China), and the leaves and stems were detached. The leaves and stems were detached and stoved in hot air oven at 55°C for 17 hr. The dried samples were grinded into flour with a drug crusher and then screened through 60 mesh sieves. The flours were packaged in airtight plastic containers prior to use.

### 2.2 Extraction and analysis of the flavonoid from *Sedum aizoon* L.

An aliquot (1.0 g) of each sample was added to 25 ml of methanol with ultrasonication for 50 min at 25°C (power 400 W, frequency 40 kHz), each extraction was repeated three times, and followed by centrifugation at 6,950  *g* for 10 min at room temperature. The obtained solution was filtered through a 0.22 μm membrane filter before direct injection into the HPLC system and stored at 4°C (Mittal, Kadyan, Gahlaut, & Dabur, 2013).

The standard solution was prepared by dissolving in methanol with the final concentrations of each compound at each concentration, respectively. All solutions filtered through a 0.22 μm membrane filter prior to analysis. These solutions were stored at 4°C for further HPLC analysis.

The flavonoids were detected at 254 nm using an HPLC system (SYNAPT G2, WATER). The compounds were separated using a reverse‐phase column (4.6 × 150 mm, 5 μm, XDB‐C18, Agilent Zorbax) with an 18‐min gradient at a flow rate of 0.8 ml/min. The mobile phase A was methanol, and the mobile phase B was 0.1% formic acid with the following gradient elution program: 0–5 min, 70%–30% A; 5–18 min, 30%–10% A. The injection volume of the sample was 10 μL, and the column temperature was 25°C.

### 2.3 Bacterial strains and culture conditions

*Aeromonas* were cultured at 30 ± 2°C on nutrient agar slant medium. Stock culture (1 ml) was passed through two successive 24 hr growth cycles at 30 ± 2°C in 9 ml of broth for liquid cultures. After 24 hr, the culture was used to inoculate 100 ml fresh broth and incubated at 30 ± 2°C for 12 hr to obtain a fresh working culture containing about 10^5^ ~ 10^6^ CFU/mL, as determined by OD_600_ (Djenane, Yangüela, Montañés, Djerbal, & Roncalés, 2011).

### 2.4 Extraction of flavonoid from *Sedum aizoon* L.

Flavonoids were extracted as previously described with minor modifications (Jarial et al., 2016). Ground powder (100 g) was extracted with 500 ml 90% ethanol for 0.5 hr at room temperature, homogenized by ultrasonication with a power of 200 W and a frequency of 40 kHz (50 min, 60°C), and incubated in a 60°C water bath for 2 hr, and then evaporated to obtain concentrate. The concentration of flavonoid concentrates was 20 mg/ml, which were stored at 4°C for later analysis.

### 2.5 Determination of the growth curves and the time–kill curves of *Aeromonas*

The growth curve and the time–kill curves of *Aeromonas* were determined according to the method of Xu et al., (2018) with slight modifications. Flavonoid concentrates were added to a final concentration of 0.675 mg/ml (1 minimum inhibitory concentration [MIC]) in the test of the measurement of growth curve, meanwhile flavonoid concentrates were 0.675 mg/ml (1 MIC) and 2.5 mg/ml (1 minimum bactericidal concentration [MBC]) in the determination of time–kill curves experiment. The MIC (1 MIC, 0.675 mg/ml) and MBC (1 MBC, 2.5 mg/ml) were based on the preliminary trial results (data not shown).

### 2.6 Determination of the electrical conductivity of the culture medium

Relative conductivity was analyzed according to the method by Diao et al., (2014). The electrical conductivity of the supernatant of the bacteria suspension treated with flavonoids was recorded as *L*_1_, and the conductivity of flavonoid aseptic ultrapure water solution in the same concentration was recorded as *L*_2_. The conductivity of the supernatant of the bacteria with aseptic water was recorded as *L*_3_, and the conductivity of the cooling solution after boiling 15 min was *L*_0_. The result was calculated according to the formula: |(*L*_1_ − *L*_2_ − *L*_3_)|/*L*_0_ × 100%.

### 2.7 Determination of OD260 value

DNA and RNA quantitation was carried out according to the procedure described by Tao, Jia, and Zhou (2014). The cells were suspended in 20 ml PBS in the presence of flavonoids at 1 MIC or 20 ml of sterile water, test and control samples were collected by centrifugations at 3,000 *g* for 10 min at 1 hr intervals for 6 hr. The absorbance was measured at 260 nm.

### 2.8 Transmission electron microscopy

*Aeromonas* treating with 1MBC (2.5 mg/ml) flavonoids and aseptic saline for 6 hr, the bacterial suspension was cleaned with PBS three times and centrifuged (10,000 *g*, 10 min). Ultrastructure observation of *Aeromonas* was determined with a transmission electron microscope (TEM, Model JEM‐1230; Hitachi) according to the procedure described by Xu et al., (2018).

### 2.9 Determination of the ATPase of *Aeromonas*

Cells were cultured to stationary phase, aliquoted into test tubes, and washed three times with PBS and then centrifuged at 3,000 *g* for 10 min, followed by resuspension in PBS. The final pellet was suspended in 20 ml of PBS. About 0.675 ml flavonoid concentrates were added to a final concentration of 0.675 mg/ml (1 MIC), all samples were incubated at 30 ± 2°C for 12 hr. Cell suspensions of the treated and control samples were adjusted to the same cell density, then the cell soluble proteins were collected. The activity of ATPase was assayed through ATP assay kit (Zou, Hu, & Chen, 2015) (Nanjing JianCheng Bioengineering Institute).

### 2.10 Determination of *Aeromonas* soluble protein content

Bacterial soluble protein determination was measured based on the method of Wang, Zou, Xie, and Xie (2014) with minor modifications. *Aeromonas* were treated by 0.675 ml flavonoid concentrates. Cell soluble proteins were collected by cell crusher. All samples were adjusted to the same cell density, suspended in loading buffer, incubated in boiling water for 10 min, and centrifuged. Supernatants were subjected to SDS‐PAGE, and then, the acrylamide gel was dyed, tested with stains, and scanned.

### 2.11 Treatment of pork meat

Lean muscle of the thigh was obtained from a local supplier (Ningbo) and then transported to the laboratory. After the aseptic treatment of pork, samples (five pieces of approximately 100 g each) were cut to obtain fifty samples, each 10 ± 0.5 g (about 3 × 3 × 2 cm^3^) and then randomly divided into two groups. Pieces in the first group were immersed into the 2 mg/ml flavonoid diluent 20 ml based on our preliminary research (data not shown) for 5 min and then drained. Pieces treated with sterile water were regarded as control. After treatment, pork pieces were placed into polyamide/polyethylene (PA/PE) composite bags (0.1 mm) and stored at −18 ± 1°C for 6 months. Colony count, pH value, total volatile basic nitrogen (TVB‐N), sensory analyses, and texture profile analysis were determined at 1‐month intervals to measure the quality of pork samples.

### 2.12 Microbial counts

Determination of the microbial load on the pork samples was measured based on the method of Shon, Eo, and Eun (2010).

### 2.13 Measurement of pH and TVB‐N

The pH value of samples was measured according to Moroney, O'Grady, O'Doherty, and Kerry (2013) using a micro pH‐meter model 7200 (Leici). TVB‐N was determined as described previously by Malle and Poumeyrol (1989), and the content was expressed as mg TVB‐N/100 g.

### 2.14 Sensory evaluation of pork

An assessment team was consisting of 12‐member panel (six males and six females) to evaluate the pork indoor at 25°C on white plastic dishes. In the storage process, the method of evaluating the pork samples in the storage process was evaluated by reference for the sensory evaluation of odor, color, viscosity, and texture and repeated three times to detect the unacceptability of the senses (≥5‐point) as the reference standard (Ligutom, Mesina, & Ganji, 1999).

### 2.15 Texture profile analysis

The frozen pork was defrosted to room temperature, and the TPA of pork was carried out by using a texture analyzer (Gao, Zhang, & Zhou, 2014). The parameters were as follows: probe P/50, pre‐test speed 2 mm/s, test speed 2 mm/s, post‐test speed 5 mm/s, strain (compression ratio) 50%, trigger force 5 g, and testing interval 5 s. Results of TPA were analyzed by TPA‐macro (Stable Micro System).

### 2.16 Statistical analyses

Statistical analysis was performed using the SPSS package program version 19.0 (SPSS Inc.). Data were analyzed by one‐way analysis of variance (ANOVA). The values are reported as mean ± standard error for all results. Mean separations were performed by Duncan's multiple range tests. Differences were considered significant at *p* < .05. At least three samples from the treatment and control groups were examined.

## 3 RESULTS

### 3.1 Chemical composition of flavonoids from *Sedum aizoon* L.

The individual flavonoid content in *S. aizoon* L. was quantitatively determined with HPLC. Flavonoids (quercetin, scutellarin, lonicerin, luteolin), flavonols (kaempferol, quercetin, rutin, isoquercitrin, isorhamnetin, trifolin), flavanols (catechin), and isoflavones (genistin) were detected in *S. aizoon* L. Kaempferol, quercetin dihydrate, and catechin were the most predominant flavonoids from *S. aizoon* L. and reached to 233.196 ± 29.675 mg/L, 233.372 ± 33.590 mg/L, and 232.238 ± 31.812 mg/L, respectively. The minor flavonoids were scutellarein and isorhamnetin, only 58.65 ± 7.900 mg/L and 59.648 ± 9.248 mg/L, respectively (Table 1).

**Table 1:** Concentration of flavonoid compounds

NO	Flavonoid compounds	Retention time (min)	Concentration (mg/L)
1	Kaempferol	13.30	233.196 ± 29.675
2	Quercetin dihydrate	11.10	234.093 ± 29.950
3	Rutin	6.84	228.174 ± 26.682
4	Luteolin	10.99	117.911 ± 16.321
5	Quercetin	8.07	232.238 ± 31.812
6	Astragalin	7.96	112.057 ± 12.639
7	Catechin	5.05	233.372 ± 33.590
8	Scutellarein	9.52	59.648 ± 9.248
9	Isoquercitrin	7.20	227.228 ± 25.468
10	Genistein	7.50	115.425 ± 13.971
11	Lonicerin	7.15	114.911 ± 13.908
12	Isorhamnetin	13.64	58.65 ± 7.900
13	Trifolin	7.68	217.458 ± 17.900

### 3.2 Effect of flavonoids from *Sedum aizoon* L. on the growth and time–kill curves in *Aeromonas*

**Figure 1:**
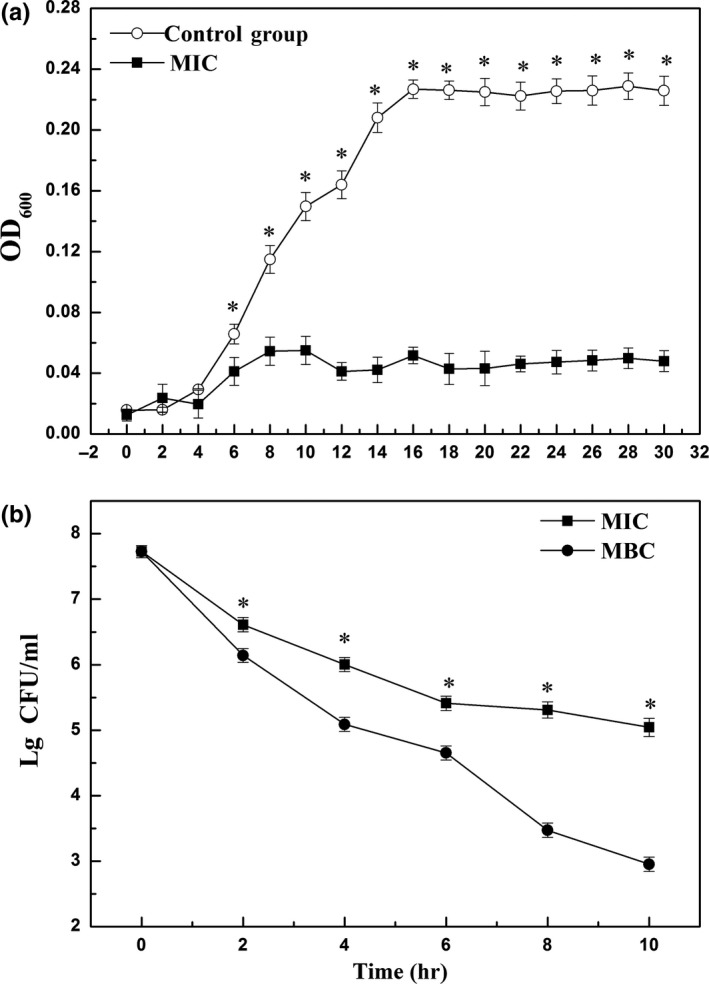
Growth (a) and time–kill (b) curves of *Aeromonas* exposed to flavonoids from *Sedum aizoon* L. Each data point is the mean of three replicate samples (mean ± standard error). Vertical bars represent standard deviation of the mean.^*^ *p* < .05 versus control group

As shown in Figure 1a, *Aeromonas* in control group got a postexponential growth phase after 4 hr incubation and reached the stationary phase at 16 hr. However, there were not remarkable changes in growth curve of the flavonoid‐treated group during the 24 hr, and *Aeromonas* maintained a relatively stable lag phase.

Figure 1b showed the reduction in the log_10_ CFU/ml of *Aeromonas* in the presence of flavonoid in MIC and MBC over 10 hr of incubation. The difference between MIC and MBC was evident during the first 2 hr and continued to expand thereafter.

### 3.3 Effect of flavonoids from *Sedum aizoon* L. on the membrane and wall in *Aeromonas*

**Figure 2:**
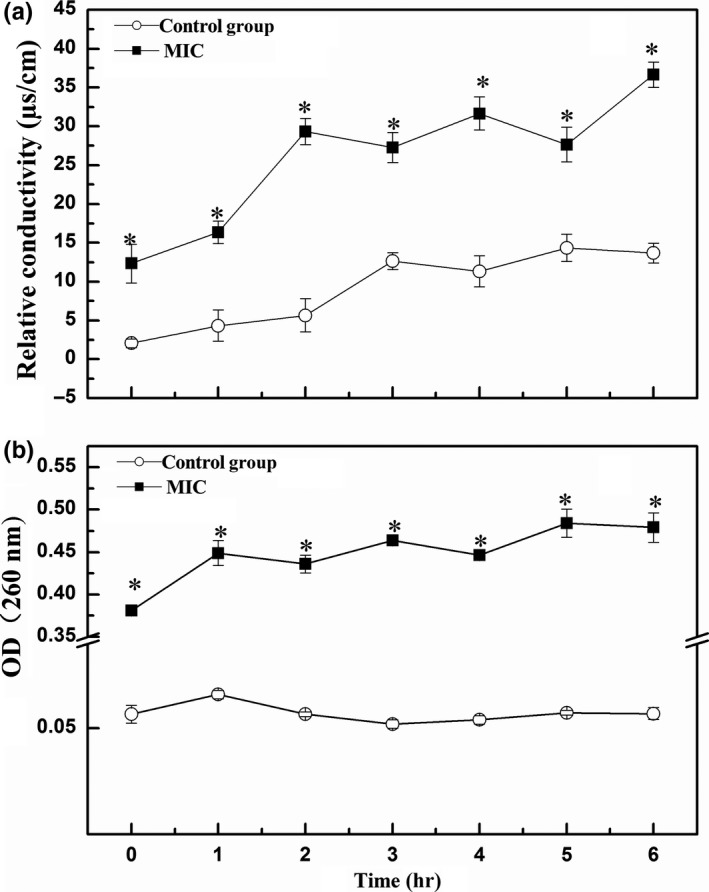
Effect of flavonoids from *Sedum aizoon* L. on the membrane of *Aeromonas* as measured by (a) relative conductivity; (b) quantity of DNA and RNA. Each data point is the mean of three replicate samples (mean ± standard error). Vertical bars represent standard deviation of the mean.^*^ *p* < .05 versus control group

Changes in membrane and wall of *Aeromonas* after flavonoid treatment were measured as electrical conductivity and the value of OD_260_. The electrical conductivity increased in both control and flavonoid‐treated samples over the 6 hr (Figure 2a), whereas the electrical conductivity in flavonoid‐treated samples was significantly higher than that of control. Meanwhile, the value of OD_260_ in suspension of flavonoid‐treated *Aeromonas* increased within the incubation. However, the control maintained the OD_260_ value at relatively low level during cultivation (Figure 2b).

### 3.4 Effect of flavonoids from *Sedum aizoon* L. on the ultrastructure in *Aeromonas*

**Figure 3:**
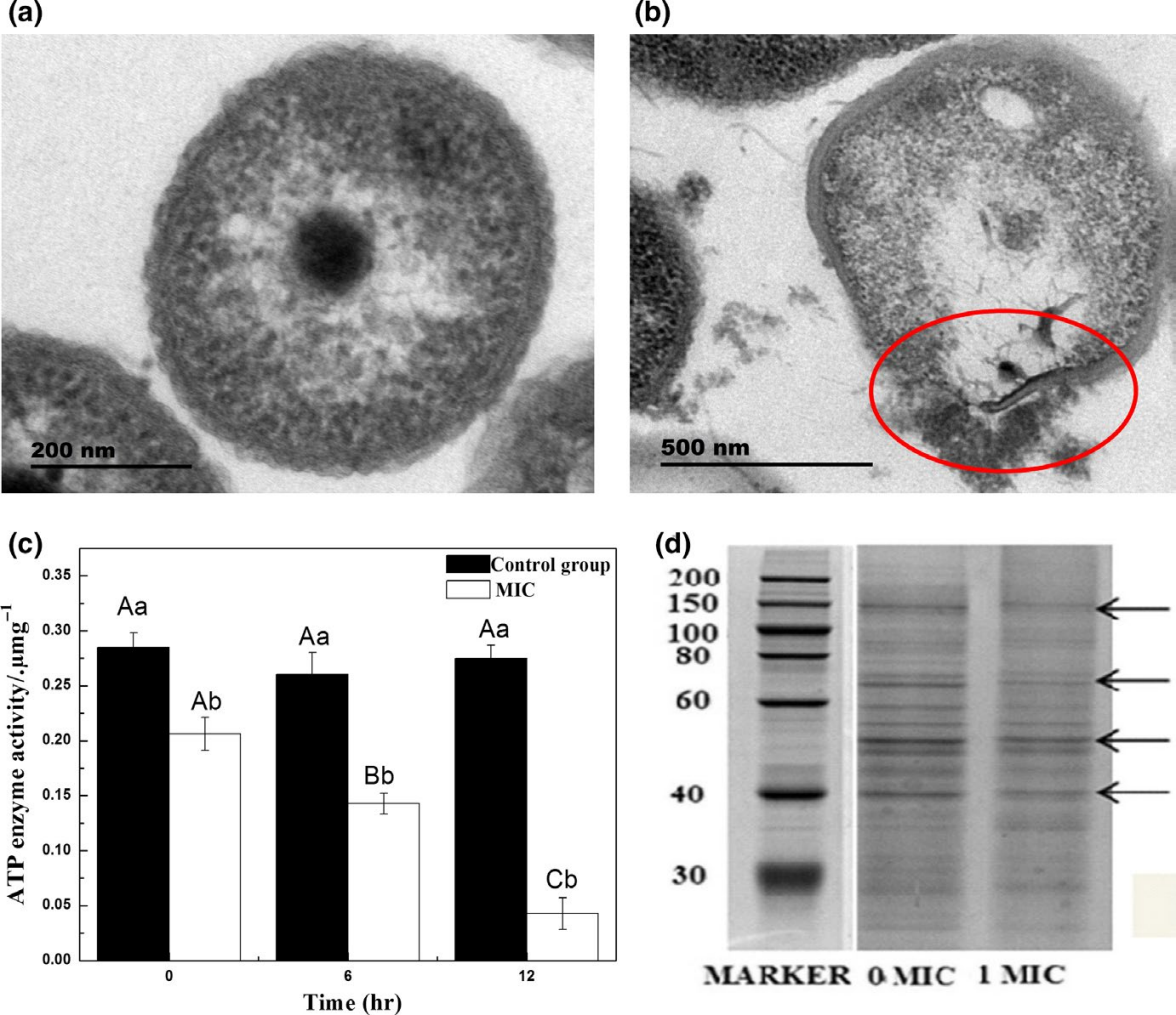
Effect of flavonoids from *Sedum aizoon* L. on the ultrastructure of *Aeromonas*. Transmission electron micrograph of (a) *Aeromonas* without flavonoids (control); (b) *Aeromonas* treated with flavonoids at minimum inhibitory concentration (MIC; 0.675 mg/ml). (Magnification is 40,000×); (c) effect of flavonoids from *S. aizoon* L. on ATPase activity of *Aeromonas*. Each data point is the mean of three replicate samples (mean ± standard error). Values not sharing a common letter are significantly different at *p* < .05; (d) SDS‐PAGE analysis of the soluble protein in *S.aizoon* L. flavonoid‐treated and mock‐treated *Aeromonas*

The ultrastructure of *Aeromonas* cells was observed by transmission electron microscopy. In the control group, complete and smooth‐shaped cells had clear membranes and homogeneity of cell matrix (Figure 3a). In contrast, flavonoid‐treated *Aeromonas* were deformed seriously, the boundaries of membranes were blurred, and a large number of unidentified extravasation was deposited on the periphery (Figure 3b).

### 3.5 Effect of flavonoids from *Sedum aizoon* L. on ATPase in *Aeromonas*

The activity of ATPase in *Aeromonas* strongly declined under flavonoid treatment (*p* < .05), while there was no obvious change observed in the control groups until the end of cultivation (Figure 3c).

### 3.6 Effect of flavonoids from *Sedum aizoon* L. on the soluble protein in *Aeromonas*

As shown in Figure 3d, the electrophoretic bands of mock‐treated *Aeromonas* were dark and clearly visible, while the bands were consistently lighter in the treated sample than in the control.

### 3.7 Effects of flavonoids from *Sedum aizoon* L. on pork quality during storage at −18°C

**Figure 4:**
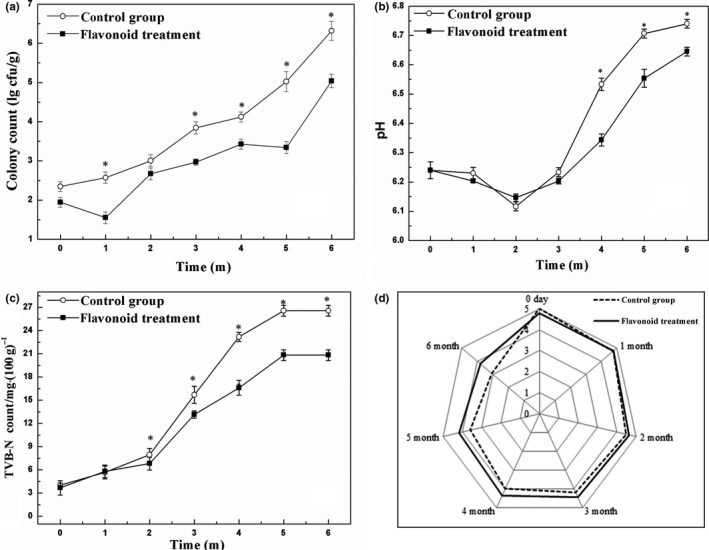
Effect of flavonoids from *Sedum aizoon* L. on microbial counts, pH and TVB‐N, and sensory evaluation of pork meat during storage at −18°C (a) microbial counts; (b) pH value; (c) TVB‐N; (d) sensory evaluation. Each data point is the mean of three replicate samples (mean ± standard error). Vertical bars represent standard deviation of the mean.^*^ *p* < .05 versus control group

The microbial counts in flavonoid‐treated pork were lower than in control (*p* < .05), particularly by month 6 when the colony on treated pork was about 100‐fold less than on the control pork (Figure 4a). The pH value in control decreased by month 2 and then increased. The pH value in the treated pork was significantly lower than the control after month 4 (*p* < .05; Figure 4b).

The TVB‐N content tended to increase gradually until 5 months, and then the expression remained unchanged till the end of the storage (Figure 4c). No statistically significant differences in TVB‐N values were observed between control and treated samples in the first 2 months of the storage. The sensory evaluation of mock and flavonoid‐treated pork increased during frozen storage (Figure 4d), which was significantly higher in the treated pork than that of control after month 3 (*p* < .05).

Table 2 showed the changes in texture characteristics of mock and flavonoid‐treated pork. The hardness in both control and treated samples maintained a moderate quantity until 3 months and then decreased obviously. However, the decline of hardness detected in control samples was higher than that in the treated ones (*p* < .05). As the storage progressed, springiness, resilience, and chewiness in frozen pork gradually decreased, however, the values were significantly higher in flavonoid‐treated samples than that in control (*p* < .05).

**Table 2:** Effects of flavonoids from *Sedum aizoon* L. on textural characteristics of freeze‐stored pork

Index	Time/month							
	Group	0	1	2	3	4	5	6
Hardness/g	Control	5,378.66 ± 393.94^Cb^	5,664.93 ± 641.15^Ab^	5,715.40 ± 473.96^Bb^	5,064.84 ± 136.93^Db^	4,648.99 ± 363.64^Eb^	4,019.33 ± 288.25^Fb^	3,321.22 ± 323.99^Gb^
	Flavonoid	5,736.95 ± 224.66^Da^	6,441.14 ± 612.94^Ba^	6,653.82 ± 645.06^Aa^	6,352.94 ± 364.19^Ba^	5,928.18 ± 488.78^Ca^	5,523.20 ± 322.90^Ea^	4,901.94 ± 409.24^Fa^
Springiness	Control	0.78 ± 0.04^Aa^	0.67 ± 0.08^Bb^	0.57 ± 0.02^Cb^	0.56 ± 0.02^Cb^	0.43 ± 0.04^Db^	0.38 ± 0.05^Eb^	0.21 ± 0.05^Fb^
	Flavonoid	0.79 ± 0.06^Ca^	0.96 ± 0.02^Aa^	0.83 ± 0.04^Ba^	0.69 ± 0.02^Da^	0.66 ± 0.03^Ea^	0.51 ± 0.01^Fa^	0.45 ± 0.01^Ga^
Resilience	Control	0.34 ± 0.01^Ba^	0.37 ± 0.01^Aa^	0.35 ± 0.01^Ba^	0.33 ± 0.01^Ba^	0.30 ± 0.01^Cb^	0.27 ± 0.01^Db^	0.25 ± 0.01^Eb^
	Flavonoid	0.34 ± 0.00^Ca^	0.38 ± 0.02^Aa^	0.36 ± 0.01^Ba^	0.32 ± 0.01^Ca^	0.33 ± 0.00^Ca^	0.31 ± 0.01^Da^	0.28 ± 0.01^Ea^
Chewiness	Control	4,117.49 ± 79.84^Ab^	3,533.00 ± 48.66^Bb^	2,896.34 ± 70.88^Cb^	2,797.17 ± 29.81^Cb^	2,019.79 ± 34.22^Db^	1,890.81 ± 65.76^Eb^	762.99 ± 65.04^Fb^
	Flavonoid	5,252.00 ± 19.07^Aa^	4,449.97 ± 43.57^Ba^	4,053.78 ± 59.46^Ca^	3,260.52 ± 29.46^Da^	3,001.98 ± 71.55^Da^	2,569.92 ± 92.57^Ea^	1,901.29 ± 78.08^Fa^

Note

Each data point is the mean of three replicate samples (mean ± standard error). Values not sharing a common letter are significantly different at *p* < .05.

## 4 DISCUSSION

It has been reported that flavonoids possess antimicrobial activity on various microorganisms (Edewor‐Kuponiyi, 2013). According to Subrata Biswas, Chowdhury, Das, Karmakar, and Shill (2011), the flavonoids from leaves and stems of *Kalanchoe pinnate* linn. (Crassulaceae) delayed the growth of bacteria. Similarly, Bensouici et al. (2016) found that the activities of *Escherichia coli*, *Pseudomonas aeruginosa,* and *Staphylococcus aureus* were obviously inhibited when treated with flavonoids from *Sedum caeruleum* L. (Crassulaceae). In our present work, the results of growth and time–kill curves showed that flavonoids from *S. aizoon* L. could prevent the logarithmic long‐term proliferation of *Aeromonas*. The higher of electrical conductivity in the flavonoid‐treated groups and absorption at 260 nm in the strains of bacteria suspended from *Aeromonas* indicated that the flavonoids from *S. aizoon* L. destroyed the physical integrity of cell membrane and led to the loss of cell constituents, which was also demonstrated with TEM observation (Figure 3b). Similar results were also found in bacterial cells treated with antibacterial compounds (He, Wu, Pan, & Xu, 2014; Tao et al., 2014; Wang, Chang, Yang, & Cui, 2015).

Intracellular ATP concentration is used to indicate the cell viability, and it can be rapidly degraded when cells die. ATPase has been reported to play an important role in microbial energy metabolism (Palmer, Kelly, & Sturtevant, 2007). Our result showed that the decline of ATPase activity was observed in the *Aeromonas* treated with flavonoid extract from *S. caeruleum* L. (Figure 3c). This observation confirmed the previous report of Chinnam et al. (2010) that flavonoids may react with the side chain of the enzyme, inhibit or even bind to ATPase and thereby result in cell apoptosis. In addition, the loss of soluble protein from flavonoid‐treated *Aeromonas* in Figure 3d may be due to flavonoids interfering with protein synthesis or the result of leakage of proteins in the damaged cell membranes (Zhao, Zhang, Hao, & Li, 2015).

Flavonoids extract from *S. aizoon* L. were demonstrated to reduce the vitality of *Aeromonas* in cell culture; however, it was more interesting when the extract was applied in frozen pork during storage. With the prolongation of storage time, the growth and reproduction speed of microorganisms accelerated and the quality of frozen meat deteriorated gradually. However, flavonoid treatment significantly inhibited the growth of microorganisms and delayed meat spoilage (Figure 4a). Meanwhile, the flavonoids from *S. aizoon* L. inhibited the change in pH value and TVB‐N count (Figure 4b,c), suggesting that it could effectively protect the freshness of pork. Natural antioxidants especially catechin have been reported to retard lipid oxidation, microbial growth, and be positively effective in maintaining sensorial quality of meat during storage (Maqsood, Abushelaibi, Manheem, Rashedi, & Kadim, 2015). In the present study, kaempferol, quercetin dihydrate, and catechin were identified as the most predominant flavonoids in *S. aizoon* L. (Table 1). Thus, we inferred that the improvement of the texture of frozen pork treated with our flavonoid extract might be due to its antioxidative ability as well, which was helpful to maintain membrane integrity of muscle fibers and retard lipid oxidation (Reihani, Tan, Huda, & Easa, 2014). Similar results were also found in pork patties treated with two natural extracts (*Rosmarinus officinalis* L. and *Melissa officinalis* L.) (Lara, Gutierrez, Timón, & Andrés, 2011).

In conclusion, flavonoids from *S. aizoon L*. exhibited antibacterial activity to *Aeromonas *in vitro, and flavonoid treatment inhibited the microbial growth and deteriorates of pork characteristics, which can be a proper natural replacement for preservatives such as nitrates, which maintains the quality of pork meat during storage and owns high consumer acceptance.

## CONFLICT OF INTEREST

The authors declare that they do not have any conflict of interest.

## ETHICAL APPROVAL

This study does not involve any human or animal testing.

## INFORMED CONSENT

Written‐informed consent was obtained from all study participants.
